# Lenvatinib recruits cytotoxic GZMK+CD8 T cells in hepatocellular carcinoma

**DOI:** 10.1097/HC9.0000000000000209

**Published:** 2023-07-17

**Authors:** Tomoharu Yamada, Naoto Fujiwara, Naoto Kubota, Yuki Matsushita, Takuma Nakatsuka, Shigeyuki Kurosaki, Tatsuya Minami, Ryosuke Tateishi, Akihiko Ichida, Junichi Arita, Kiyoshi Hasegawa, Kazuhiko Koike, Mitsuhiro Fujishiro, Hayato Nakagawa

**Affiliations:** 1 Department of Gastroenterology, Graduate School of Medicine, The University of Tokyo, Tokyo, Japan; 2 Department of Gastroenterology and Hepatology, Mie University, Tsu, Japan; 3 Department of Internal Medicine, Division of Digestive and Liver Diseases, University of Texas Southwestern Medical Center, Dallas, Texas, USA; 4 Department of Surgery, Hepato-Biliary-Pancreatic Surgery Division, Graduate School of Medicine, The University of Tokyo, Tokyo, Japan; 5 Department of Gastroenterological Surgery, Akita University Graduate School of Medicine, Akita, Japan

## Abstract

Lenvatinib was expected to enhance the effect of immune checkpoint inhibitors (ICIs) for unresectable HCC; however, their combination therapy failed to show the synergy in the phase III clinical trial.

**Methods:**

To elucidate lenvatinib-induced molecular modulation, we performed bulk RNA-sequencing and digital spatial profiling of 5 surgically resected human HCC specimens after lenvatinib treatment and 10 matched controls without any preceding therapy.

**Findings:**

Besides its direct antitumor effects, lenvatinib recruited cytotoxic GZMK+CD8 T cells in intratumor stroma by CXCL9 from tumor-associated macrophages, suggesting that lenvatinib-treated HCC is in the so-called excluded condition that can diminish ICI efficacy.

## Graphical abstract

**Figure:**
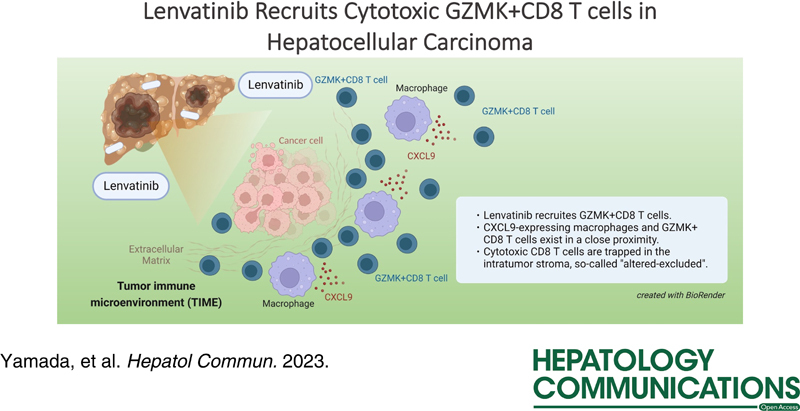

## INTRODUCTION

HCC, representing >80% of primary liver cancer, is the fourth leading cause of cancer-related death worldwide.^1^ While an early-stage HCC can be curatively treated, 18% of HCC is diagnosed at a later stage with limited therapeutic options, resulting in its dismal prognosis (<15% of 5-y survival).^1^ Lenvatinib, targeting multiple kinase receptors including VEGF receptors 1–3, FGF receptors 1–4, platelet-derived growth factor receptor α, *KIT*, and *RET*, is a therapy with a proven survival benefit for unresectable HCC.^2^ Given the target molecules of lenvatinib, its combination with an immune checkpoint inhibitor (ICI) was also expected to synergistically enhance the antitumor response as observed in the combination therapy of anti-PD-L1 atezolizumab with anti-VEGF bevacizumab.^3^ However, compared with lenvatinib alone, the combination therapy with anti-PD-1 pembrolizumab failed to show the additive prognostic benefit in patients with advanced HCC in the phase III trial despite promising results in experimental models (NCT03713593).^4^ Thus, elucidating mechanisms of action by lenvatinib in humans would be needed for successful translation of experimental findings and further improvement of its efficacy in clinical practice. To reveal lenvatinib-induced molecular modulation, we performed multilayer transcriptome analyses of surgically resected human HCC samples after lenvatinib treatment as neoadjuvant therapy in the phase II clinical trial together with matched control HCCs without any preceding therapy (LENS-HCC trial, jRCTs031190057).

## METHODS

### Patients

This study consisted of 5 patients who underwent surgical resection for HCC after neoadjuvant lenvatinib treatment in the phase II clinical trial (LENS-HCC) and 10 matched HCC patients without any preceding treatment between April 2018 and July 2020 at the University of Tokyo Hospital. The matched control patients were selected according to age, sex, tumor size, and etiology.

### Transcriptome profiling

Total RNA extracted from human frozen HCC tissues by ISOGEN (NIPPON GENE) was subjected to RNA-seq library preparation with the TruSeq Stranded mRNA Library Prep (Illumina) and sequenced with NovaSeq. 6000 system (Illumina) according to the manufacturer’s instructions. The dataset is publicly available at the NCBI GEO (accession number, GSE223201).

### Digital spatial profiling (DSP)

The 5 lenvatinib-treated HCCs and 1 metastatic lymph node were subjected to the DSP. In total, 72 regions of interest were included. The formalin-fixed paraffin-embedded slides were deparaffinized and stained with immunofluorescent antibodies to detect tumor cells (KRT8/18), immune cells (CD45), T cells (CD3), and DNA to visualize the morphology for regions of interest. Once the staining was completed, slides were loaded onto a GeoMx DSP instrument and scanned to produce digital immunofluorescence images.

### Statistical analysis

Continuous variables were compared by nonparametric Wilcoxon rank-sum test and Spearman correlation. All bioinformatic and biostatistical analyses were performed using R statistical language (www.r-project.org).

The details for patient selection and multilayer transcriptome, histological, and statistical analyses were described in Supplemental Methods, http://links.lww.com/HC9/A409.

## RESULTS

**FIGURE 1:**
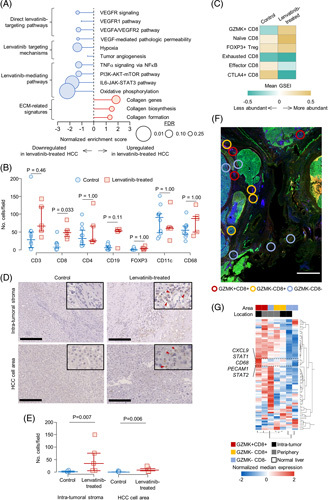
Lenvatinib recruits GZMK + CD8 cells into HCC. (A) Modulated pathways in lenvatinib-treated HCC compared with matched control HCCs by bulk transcriptome profiling. (B) Accumulated immune cells in lenvatinib-treated and control HCC. *p*-values were corrected by Bonferroni correction. (C) Gene signature–based immune cell characterization. (D) Accumulated GZMK+CD8 T cells in the intratumor stroma and HCC cell area. Arrowheads indicate GZMK+CD8 T cells. Scale bar, 250 μm. (E) Number of GZMK+CD8 T cells in the intratumor stroma and HCC cell area. (F) Representative image of region selection in the DSP. Scale bar, 3 mm. (G) Expression pattern of immune-related genes in GZMK+CD8+T-cell-rich, GZMK-CD8+T cell–rich, and GZMK-CD8- regions. Abbreviations: CXCL9, C-X-C motif chemokine ligand 9; DSP, digital spatial profiling; FDR, false discovery rate; GSEI, gene set enrichment index, GZMK, granzyme K; IQR, interquartile range; VEGFR, VEGRF receptor.

The study design and patient demographics are summarized in Supplemental Figure S1A, B, http://links.lww.com/HC9/A409. Median dosing and washout period were 43 (interquartile range, 28–62) and 16 (interquartile range, 13–20) days, respectively, indicating that these samples were appropriate for analyzing the ongoing effects of lenvatinib on HCC. Among 5 lenvatinib-treated HCCs, 3 (60%) achieved partial response (Supplemental Figure S1B, http://links.lww.com/HC9/A409). Molecular pathway analyses revealed that the VEGFR pathway, especially the VEGFR2 pathway, was downregulated together with other HCC drivers in lenvatinib-treated HCC, whereas no significant modulation of other potential lenvatinib targets, such as FGF receptor 4 pathway, was observed (Figure 1A, Supplemental Table S1, http://links.lww.com/HC9/A409). VEGF is known to suppress antitumor immunity through various mechanisms,^5^ among which VEGF-mediated permeability and hypoxia pathways were significantly downregulated in lenvatinib-treated HCC, suggesting that lenvatinib might improve aberrant tumor vascularity and promoted immune cell infiltration that can exert antitumor immunity. Indeed, lenvatinib-treated HCC pathologically exhibited more frequent immune cell infiltration compared with control HCC (100% and 50% for lenvatinib-treated and control HCC, respectively) (Supplemental Figure S1B, http://links.lww.com/HC9/A409). Besides, lenvatinib-treated HCC showed upregulated fibrosis-related pathways, especially among patients who achieved a partial response (Supplemental Figure S1C, http://links.lww.com/HC9/A409), suggesting more abundant extracellular matrix (ECM) deposition (Figure 1A). Next, to evaluate which immune cell types mainly increased in lenvatinib-treated HCC, we performed immunohistochemistry for immune cell surface markers (Figure 1B). Of note, the main source of increased immune cell infiltration was CD8 T cells, with incremental trends in the other cell types, such as B cells. Furthermore, gene signature–based subpopulation characterization of CD8 T cells suggested that granzyme K (GZMK)+CD8 T cells increased in lenvatinib-treated HCC (Figure 1C). Subsequent immunohistochemistry evaluation confirmed that GZMK+CD8 T cells were significantly recruited in lenvatinib-treated HCC compared with control HCC, especially in the intratumor stroma (Figure 1D, E). There was no association between therapeutic response and the abundance of CD8 T-cell infiltration (Figure S1D, http://links.lww.com/HC9/A409). Collectively, these findings suggested that lenvatinib increased GZMK+CD8 T-cell infiltration by improving tumor-aberrant vascularity through downregulated VEGFR pathways.

A recent large-scale single-cell RNA-sequence study showed that GZMK+CD8 T cells in liver cancer are cytotoxic rather than exhausted.^6^ Consistently, higher *GZMK* expression in HCC was associated with a favorable prognosis in multiple HCC cohorts (Supplemental Figure S1E, http://links.lww.com/HC9/A409). To further characterize GZMK+CD8 T-cell–rich microenvironment in lenvatinib-treated HCC, we performed a spatial transcriptome analysis using Digital Spatial Profiling technology (NanoString) (Figure 1F, Supplemental Figure S1F, http://links.lww.com/HC9/A409).^7^ GZMK+CD8 T cells coexisted with other immune cells as evidenced by high immune-related gene expressions, irrespective of intratumoral and peritumoral regions. Interestingly, differential expression analysis revealed that C-X-C motif chemokine ligand 9 (*CXCL9*) (encoding CXCL9) and *CD68* (encoding CD68) showed significantly higher expression more specifically in intratumor GZMK+CD8 T-cell–rich regions, suggesting that CXCL9-expressing tumor-associated macrophages (TAMs) and GZMK+CD8 T cells exist in close proximity (Figure 1G). Dual immunofluorescence staining and single-cell RNA-sequencing analyses confirmed that CXCL9 was almost exclusively expressed in macrophages in HCC (Supplemental Figure S1G, H, http://links.lww.com/HC9/A409). CXCL9-expressing TAMs have been well-recognized to promote T-cell infiltration in various tumors.^8^ Consistently, the expression levels of *CXCL9* and *GZMK* were strongly correlated in HCCs from multiple independent cohorts (Supplemental Figure S1I, http://links.lww.com/HC9/A409).

## DISCUSSION

In this study, multilayer transcriptome analyses revealed that lenvatinib recruited cytotoxic GZMK+CD8 T cells by improving aberrant vascularity and its direct antitumor effect. However, the majority of these cytotoxic CD8 T cells appeared to be trapped in the intratumor stroma, indicating that lenvatinib-treated HCC might be “altered-excluded” due to physical barriers by ECM rather than exhausted.^9^ Altered-excluded tumors are generally believed to be less responsive to ICI,^10^ which might lead to failure to show the additive effects of their combination therapy. Various factors can drive tumors toward altered-excluded status.^10^ Given improved aberrant vascularity and T-cell chemotaxis by *CXCL9* after lenvatinib treatment, ECM might be a potential target to tackle the limited efficacy of combination therapy of lenvatinib and ICI. Recently, anti-ECM compounds, such as fresolimumab (collagen synthesis inhibitor) and simtuzumab (collagen cross-linking inhibitor), have been actively explored for cancer treatment in early-stage clinical trials.^11^ Adding these anti-ECM drugs to the combination therapy of lenvatinib and ICI might enable cytotoxic GZMK+CD8 T cells to more easily penetrate inside the HCC cell area, eventually leading to improved drug efficacy and favorable patient prognosis. Higher resolution spatial transcriptome analyses would enable us to infer the cell-cell interaction between GZMK+CD8 T cells and CXCL9-expressing TAMs/fibroblasts.

## Supplementary Material
