# Supplementary material for: Lenvatinib recruits cytotoxic GZMK+CD8 T cells in hepatocellular carcinoma
Source: Hepatol Commun. 2023 Jul 17;7(8):e0209. doi: 10.1097/HC9.0000000000000209 (PMC10351952; doi:10.1097/HC9.0000000000000209)
Supplement: Supplementary file 1 [file hc9-7-e0209-s001.docx]

**Supplementary Methods**

**Patients**

This study consisted of 5 patients who underwent surgical resection for hepatocellular carcinoma (HCC) after neoadjuvant lenvatinib treatment in the phase II clinical trial (LENS-HCC trial, jRCTs031190057) and 10 matched HCC patients without any preceding treatment between April 2018 and July 2020 at the University of Tokyo Hospital (**Figure S1B**). The matched control patients were selected according to age, sex, tumor size, and etiology. HCC samples were collected and snap-frozen immediately after HCC resection and stored at -80°C until use. The efficacy of neoadjuvant lenvatinib was radiologically evaluated by dynamic computed tomography or magnetic resonance imaging according to the modified Response Evaluation Criteria in Solid Tumors guidelines.^[1]^ All tissue samples were collected and used under the approval from the University of Tokyo Medical Research Center Ethics Committee (Approval Number: G10144). All patients provided written informed consent.

**Immunohistochemistry**

Five-micron thick formalin-fixed paraffin-embedded (FFPE) sections were deparaffinized with Histo-Clear (National Diagnostics) and rehydrated through graded alcohol. Target Retrieval Solution S1700 (Dako) at 95°C for 30 min was used for heat-mediated antigen retrieval, and then the tissue sections were incubated overnight at 4°C with the primary antibodies. Biotinylated secondary antibodies (PharMingen) were added and incubated for 20 min at room temperature. The sections were incubated with streptavidin-horseradish peroxidase (PharMingen) for 30 minutes, developed with 3,3’-diaminobenzidine (DAB) substrate (Vector Laboratories), and counterstained with hematoxylin. Anti-CD3 antibody (Cell Signaling Technology, 85061), anti-CD8α antibody (Cell Signaling Technology, 70306), anti-CD4 antibody (abcam, ab203034), anti-CD19 antibody (Cell Signaling Technology, 90176), anti-FoxP3 antibody (Cell Signaling Technology, D2W8E), anti-CD11c antibody (Cell Signaling Technology, 45581), anti-CD68 antibody (Cell Signaling Technology, 76437), and anti-GZMK antibody (R&D, MAB10216) were used. The slides were scanned using the NanoZoomer Digital Pathology slide scanning system (Hamamatsu Photonics) in bright-field. The number of positive cells in 5 randomly selected fields (500,000 μm^2^) were counted using the ImageJ separately and the median count of the 5 fields was used as representative abundance of positive cells in individual samples. For immunofluorescence, slides were incubated with primary antibodies, and subsequently, secondary antibodies conjugated with Alexa Fluor 488 or 555 (Invitrogen) were applied. Anti-CD68 antibody (abcam, ab201340) and anti-CXCL9 antibody (Cell Signaling Technology, 30327) were used.

**Transcriptome profiling**

Total RNA extracted from human frozen HCC tissues by ISOGEN (NIPPON GENE) were subjected to RNA-seq library preparation with the TruSeq Stranded mRNA Library Prep (Illumina) and sequenced with NovaSeq 6000 system (Illumina) according to the manufacturer’s instructions. The raw sequencing reads were mapped to the reference genome (hg19) using the STAR aligner,^[2]^ converted into transcript abundance data by the Subread package featureCounts,^[3]^ and normalized as the relative log expression (RLE) by the DESeq2 package.^[4]^ Poor-quality profiles were identified by inter-sample correlation <0.7 and excluded, and genes with low inter-sample variation (coefficient of variation <0.01) and expressed in <50% of samples were filtered out in subsequent general analyses such as exploration of molecular pathway dysregulation. The dataset is publicly ava ilable at the NCBI GEO (accession number, GSE223201). For evaluating cell-type specificity of CXCL9 in HCC, we re-analyzed a single-cell RNA-sequencing dataset extracted from Gene Expression Omnibus (GSE151530).^[5]^

**Assessment of molecular signature**

Induction or suppression of molecular signatures of pathways and cell types were determined using comprehensive gene set collections of various molecular pathways in the Molecular Signature Database^[6]^ and gene sets from literature by gene set enrichment analysis^[7]^ using fgsea R package and visualized as gene set enrichment index (GSEI).^[8]^ The signatures of CD8 subpopulation were extracted from the single-cell RNA-sequence study of HCC.^[9]^

**Prognostic association of GZMK expression**

Large-scale preprocessed transcriptome datasets with prognostic information were extracted from the public domain.^[10-11]^ High *GZMK* level was defined as higher than median in each cohort.

**Digital Spatial Profiling (DSP)**

The 5 lenvatinib-treated HCCs as well as one metastatic lymph node were subjected to the DSP. In total, 72 regions of interest (ROIs) were included. The FFPE slides were deparaffinized and stained with immunofluorescent antibodies to detect tumor cells (KRT8/18), immune cells (CD45), T cells (CD3), and DNA to visualize the morphology for ROIs. Once the staining was completed, slides were loaded onto a GeoMx DSP instrument and scanned to produce digital immunofluorescence images. Then we selected twelve ROIs, measuring 500μm in diameter, per slide. ROIs were segmented in three categories based on tissue compartments by fluorescence localization: “Intra-tumor”, “Periphery”, and “Normal liver”. The 73-plex Immuno-Oncology panel was applied and antibody oligos were photocleaved within the selected ROIs by ultra-violet illumination and collected for nCounter Analysis System. Digital counts from barcodes corresponding to RNA probes were first normalized to internal spike-in controls developed by the External RNA Controls Consortium (ERCC). The raw data were then normalized to a panel of housekeeping RNA. Sequential sections of FFPE slides were immunostained with anti-CD8 antibody and anti-GZMK antibody.

**Statistical analysis**

Continuous variables were compared by non-parametric Wilcoxon rank-sum test and Spearman’s correlation. Correction for multiple hypothesis testing by Bonferroni correction was applied as needed. For time-to-event analyses, prognostic association of high *GZMK* level was assessed using Kaplan-Meier curves and the log-rank test. A two-tailed p-value <0.05 was regarded as statistically significant. All bioinformatic and biostatistical analyses were performed using R statistical language (www.r-project.org).

**REFERENCES**

1. Lencioni R, Llovet JM. Modified RECIST (mRECIST) assessment for hepatocellular carcinoma. Semin Liver Dis 2010;30:52-60.

2. Dobin A, Davis CA, Schlesinger F, Drenkow J, Zaleski C, Jha S, Batut P, et al. STAR: ultrafast universal RNA-seq aligner. Bioinformatics 2013;29:15-21.

3. Liao Y, Smyth GK, Shi W. featureCounts: an efficient general purpose program for assigning sequence reads to genomic features. Bioinformatics 2014;30:923-930.

4. Love MI, Huber W, Anders S. Moderated estimation of fold change and dispersion for RNA-seq data with DESeq2. Genome Biol 2014;15:550.

5. Ma L, Wang L, Khatib SA, Chang CW, Heinrich S, Dominguez DA, Forgues M, et al. Single-cell atlas of tumor cell evolution in response to therapy in hepatocellular carcinoma and intrahepatic cholangiocarcinoma. J Hepatol 2021;75:1397-1408.

6. Liberzon A, Subramanian A, Pinchback R, Thorvaldsdóttir H, Tamayo P, Mesirov JP. Molecular signatures database (MSigDB) 3.0. Bioinformatics 2011;27:1739-1740.

7. Subramanian A, Tamayo P, Mootha VK, Mukherjee S, Ebert BL, Gillette MA, Paulovich A, et al. Gene set enrichment analysis: a knowledge-based approach for interpreting genome-wide expression profiles. Proc Natl Acad Sci U S A 2005;102:15545-15550.

8. Fujiwara N, Kubota N, Crouchet E, Koneru B, Marquez CA, Jajoriya AK, Panda G, et al. Molecular signatures of long-term hepatocellular carcinoma risk in nonalcoholic fatty liver disease. Sci Transl Med 2022;14:eabo4474.

9. Zheng C, Zheng L, Yoo JK, Guo H, Zhang Y, Guo X, Kang B, et al. Landscape of Infiltrating T Cells in Liver Cancer Revealed by Single-Cell Sequencing. Cell 2017;169:1342-1356.e1316.

10. Comprehensive and Integrative Genomic Characterization of Hepatocellular Carcinoma. Cell 2017;169:1327-1341.e1323.

11. Roessler S, Jia HL, Budhu A, Forgues M, Ye QH, Lee JS, Thorgeirsson SS, et al. A unique metastasis gene signature enables prediction of tumor relapse in early-stage hepatocellular carcinoma patients. Cancer Res 2010;70:10202-10212.

**
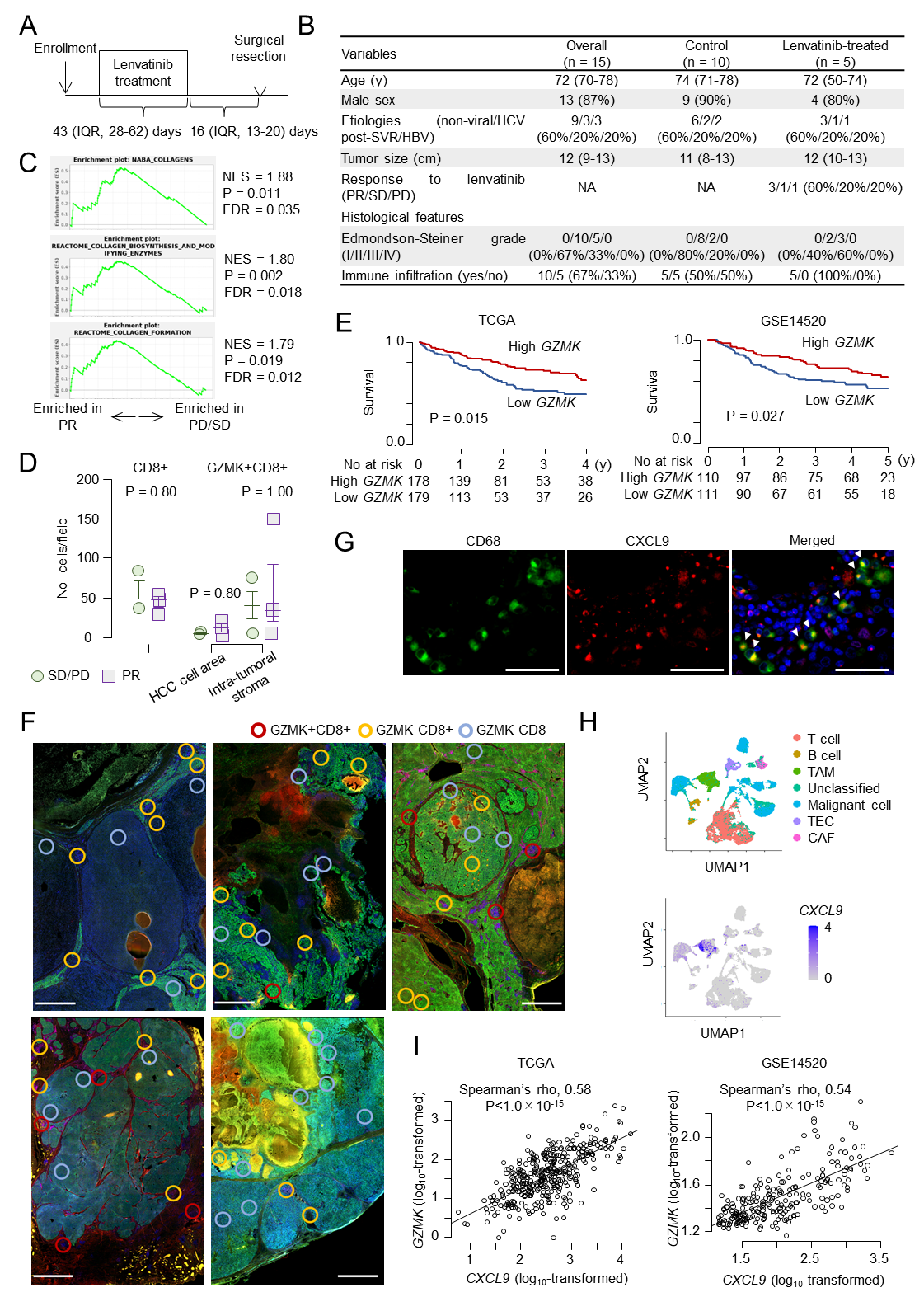
**

**Figure S1.** (A) Study design. (B) Patient demographics. (C) Enrichment of collagen-related gene signatures in HCC that achieved partial response. (D) The association between response to lenvatinib and CD8 T cells infiltration. (E) Prognostic associations of high GZMK expression in HCC. (F) ROI selection for DSP analysis. Scale bar, 3mm. (G) CXCL9+ macrophage in lenvatinib-treated HCC. Scale bar, 50μm. (H) *CXCL9* was exclusively expressed in a subset of tumor-associated macrophage in HCC. (I) Correlatoins between *GZMK* and *CXCL9* expressions in mutliple HCC cohorts.

IQR, interquartile range; HCV, hepatitis C virus; SVR, sustained virologic response; HBV, hepatitis B virus; PR, partial response; SD, stable disease; PD, progressive disease; NES, normalized enrichment score, FDR, false discovery rate; GZMK, granzyme K HCC, hepatocellular carcinoma; TCGA, the Cancer Genome Atlas; TAM, tumor-associated macrophage; TEC, tumor-associated endothelial cells; CAF, cancer-associated fibroblast; UMAP, uniform manifold approximation and projection.

**Table S1. Molecular pathways associated with lenvatinib-treated HCC.**

| Gene set database | Gene set | NES | p | FDR | Gene set | NES | p | FDR |
| --- | --- | --- | --- | --- | --- | --- | --- | --- |
|  | Enriched in lenvatinib-treated HCC |  |  |  | Enriched in control HCC |  |  |  |
| Hallmark | G2M_CHECKPOINT | 2.55 | 0.005 | 0.017 | OXIDATIVE_PHOSPHORYLATION | -2.21 | 0.001 | 0.009 |
| (Meta-analysis pathway targets) | E2F_TARGETS | 2.37 | 0.005 | 0.017 | XENOBIOTIC_METABOLISM | -2.10 | 0.001 | 0.009 |
|  | MITOTIC_SPINDLE | 1.62 | 0.005 | 0.017 | ALLOGRAFT_REJECTION | -1.98 | 0.001 | 0.009 |
|  | EPITHELIAL_MESENCHYMAL_TRANSITION | 1.58 | 0.005 | 0.017 | ADIPOGENESIS | -1.91 | 0.001 | 0.009 |
|  |  |  |  |  | FATTY_ACID_METABOLISM | -1.89 | 0.001 | 0.009 |
|  |  |  |  |  | COMPLEMENT | -1.88 | 0.001 | 0.009 |
|  |  |  |  |  | IL6_JAK_STAT3_SIGNALING | -1.81 | 0.003 | 0.015 |
|  |  |  |  |  | COAGULATION | -1.76 | 0.001 | 0.009 |
|  |  |  |  |  | BILE_ACID_METABOLISM | -1.63 | 0.004 | 0.016 |
|  |  |  |  |  | INFLAMMATORY_RESPONSE | -1.56 | 0.002 | 0.015 |
|  |  |  |  |  | MTORC1_SIGNALING | -1.51 | 0.004 | 0.016 |
|  |  |  |  |  | INTERFERON_GAMMA_RESPONSE | -1.51 | 0.004 | 0.016 |
|  |  |  |  |  | ESTROGEN_RESPONSE_EARLY | -1.47 | 0.010 | 0.029 |
|  |  |  |  |  | PEROXISOME | -1.45 | 0.020 | 0.053 |
|  |  |  |  |  | KRAS_SIGNALING_UP | -1.42 | 0.012 | 0.034 |
|  |  |  |  |  | ANDROGEN_RESPONSE | -1.37 | 0.047 | 0.098 |
|  |  |  |  |  | KRAS_SIGNALING_DN | -1.36 | 0.047 | 0.098 |
|  |  |  |  |  | HYPOXIA | -1.36 | 0.026 | 0.064 |
|  |  |  |  |  | APOPTOSIS | -1.35 | 0.040 | 0.091 |
|  |  |  |  |  | INTERFERON_ALPHA_RESPONSE | -1.35 | 0.054 | 0.105 |
|  |  |  |  |  |  |  |  |  |
| Biocarta | SM_PATHWAY | 1.96 | 0.005 | 0.107 | CTL_PATHWAY | -2.01 | 0.002 | 0.107 |
| (Curated pathway members) | CIRCADIAN_PATHWAY | 1.83 | 0.004 | 0.107 | COMP_PATHWAY | -1.98 | 0.003 | 0.107 |
|  | CDC25_PATHWAY | 1.81 | 0.007 | 0.126 | TCRA_PATHWAY | -1.98 | 0.003 | 0.107 |
|  | ATM_PATHWAY | 1.71 | 0.013 | 0.187 | IL12_PATHWAY | -1.91 | 0.003 | 0.107 |
|  |  |  |  |  | CLASSIC_PATHWAY | -1.90 | 0.002 | 0.107 |
|  |  |  |  |  | MONOCYTE_PATHWAY | -1.89 | 0.003 | 0.107 |
|  |  |  |  |  | EIF_PATHWAY | -1.85 | 0.003 | 0.107 |
|  |  |  |  |  | ASBCELL_PATHWAY | -1.84 | 0.005 | 0.107 |
|  |  |  |  |  | BBCELL_PATHWAY | -1.81 | 0.005 | 0.107 |
|  |  |  |  |  | LYMPHOCYTE_PATHWAY | -1.79 | 0.005 | 0.107 |
|  |  |  |  |  | GRANULOCYTES_PATHWAY | -1.76 | 0.010 | 0.142 |
|  |  |  |  |  | NO2IL12_PATHWAY | -1.75 | 0.005 | 0.107 |
|  |  |  |  |  | LECTIN_PATHWAY | -1.75 | 0.006 | 0.117 |
|  |  |  |  |  | NEUTROPHIL_PATHWAY | -1.74 | 0.003 | 0.107 |
|  |  |  |  |  | CSK_PATHWAY | -1.73 | 0.006 | 0.117 |
|  |  |  |  |  | BLYMPHOCYTE_PATHWAY | -1.71 | 0.010 | 0.142 |
|  |  |  |  |  | TCAPOPTOSIS_PATHWAY | -1.68 | 0.008 | 0.131 |
|  |  |  |  |  | NKT_PATHWAY | -1.67 | 0.014 | 0.189 |
|  |  |  |  |  | THELPER_PATHWAY | -1.65 | 0.016 | 0.195 |
|  |  |  |  |  | TH1TH2_PATHWAY | -1.65 | 0.016 | 0.195 |
|  |  |  |  |  |  |  |  |  |
| KEGG | RIBOSOME | 2.10 | 0.004 | 0.023 | PORPHYRIN_AND_CHLOROPHYLL_METABOLISM | -2.54 | 0.001 | 0.011 |
| (Curated pathway members) | HOMOLOGOUS_RECOMBINATION | 1.66 | 0.021 | 0.068 | ASCORBATE_AND_ALDARATE_METABOLISM | -2.39 | 0.001 | 0.011 |
|  | DNA_REPLICATION | 1.59 | 0.025 | 0.080 | STEROID_HORMONE_BIOSYNTHESIS | -2.25 | 0.001 | 0.011 |
|  | VASCULAR_SMOOTH_MUSCLE_CONTRACTION | 1.46 | 0.008 | 0.036 | RETINOL_METABOLISM | -2.22 | 0.001 | 0.011 |
|  | RIBOFLAVIN_METABOLISM | 1.45 | 0.077 | 0.177 | PENTOSE_AND_GLUCURONATE_INTERCONVERSIONS | -2.22 | 0.002 | 0.011 |
|  | MATURITY_ONSET_DIABETES_OF_THE_YOUNG | 1.44 | 0.069 | 0.166 | AUTOIMMUNE_THYROID_DISEASE | -2.21 | 0.001 | 0.011 |
|  | DILATED_CARDIOMYOPATHY | 1.39 | 0.051 | 0.130 | DRUG_METABOLISM_OTHER_ENZYMES | -2.20 | 0.001 | 0.011 |
|  | LONG_TERM_POTENTIATION | 1.35 | 0.085 | 0.190 | OXIDATIVE_PHOSPHORYLATION | -2.20 | 0.001 | 0.011 |
|  | GNRH_SIGNALING_PATHWAY | 1.34 | 0.045 | 0.121 | TYPE_I_DIABETES_MELLITUS | -2.20 | 0.001 | 0.011 |
|  | CELL_CYCLE | 1.30 | 0.048 | 0.125 | CITRATE_CYCLE_TCA_CYCLE | -2.18 | 0.002 | 0.011 |
|  | HEDGEHOG_SIGNALING_PATHWAY | 1.28 | 0.106 | 0.225 | GRAFT_VERSUS_HOST_DISEASE | -2.15 | 0.002 | 0.011 |
|  | OOCYTE_MEIOSIS | 1.20 | 0.118 | 0.246 | ALLOGRAFT_REJECTION | -2.08 | 0.002 | 0.011 |
|  |  |  |  |  | PARKINSONS_DISEASE | -2.06 | 0.001 | 0.011 |
|  |  |  |  |  | PROPANOATE_METABOLISM | -2.00 | 0.001 | 0.011 |
|  |  |  |  |  | ARGININE_AND_PROLINE_METABOLISM | -1.98 | 0.001 | 0.011 |
|  |  |  |  |  | ANTIGEN_PROCESSING_AND_PRESENTATION | -1.91 | 0.001 | 0.011 |
|  |  |  |  |  | COMPLEMENT_AND_COAGULATION_CASCADES | -1.88 | 0.001 | 0.011 |
|  |  |  |  |  | AMINOACYL_TRNA_BIOSYNTHESIS | -1.86 | 0.003 | 0.018 |
|  |  |  |  |  | GLYCINE_SERINE_AND_THREONINE_METABOLISM | -1.86 | 0.005 | 0.024 |
|  |  |  |  |  | DRUG_METABOLISM_CYTOCHROME_P450 | -1.85 | 0.001 | 0.011 |
|  |  |  |  |  |  |  |  |  |
| Reactome | POLO_LIKE_KINASE_MEDIATED_EVENTS | 2.24 | 0.003 | 0.103 | GLUCURONIDATION | -2.38 | 0.002 | 0.103 |
| (Curated pathway members) | RESOLUTION_OF_D_LOOP_STRUCTURES | 2.23 | 0.003 | 0.103 | THE_CITRIC_ACID_TCA_CYCLE_AND_RESPIRATORY_ELECTRON_TRANSPORT | -2.26 | 0.001 | 0.103 |
|  | EUKARYOTIC_TRANSLATION_ELONGATION | 2.21 | 0.004 | 0.103 | COMPLEMENT_CASCADE | -2.23 | 0.001 | 0.103 |
|  | G1_S_SPECIFIC_TRANSCRIPTION | 2.21 | 0.003 | 0.103 | INITIAL_TRIGGERING_OF_COMPLEMENT | -2.23 | 0.003 | 0.103 |
|  | RESOLUTION_OF_D_LOOP_STRUCTURES_THROUGH_SYNTHESIS_DEPENDENT_STRAND_ANNEALING_SDSA | 2.20 | 0.003 | 0.103 | RESPIRATORY_ELECTRON_TRANSPORT_ATP_SYNTHESIS_BY_CHEMIOSMOTIC_COUPLING_AND_HEAT_PRODUCTION_BY_UNCOUPLING_PROTEINS | -2.19 | 0.001 | 0.103 |
|  | G2_M_DNA_DAMAGE_CHECKPOINT | 2.09 | 0.003 | 0.103 | RESPIRATORY_ELECTRON_TRANSPORT | -2.17 | 0.001 | 0.103 |
|  | RESPONSE_OF_EIF2AK4_GCN2_TO_AMINO_ACID_DEFICIENCY | 2.09 | 0.004 | 0.103 | HEME_DEGRADATION | -2.15 | 0.002 | 0.103 |
|  | MITOTIC_PROMETAPHASE | 2.02 | 0.006 | 0.119 | METABOLISM_OF_PORPHYRINS | -2.14 | 0.003 | 0.103 |
|  | HOMOLOGY_DIRECTED_REPAIR | 2.00 | 0.004 | 0.103 | MITOCHONDRIAL_FATTY_ACID_BETA_OXIDATION | -2.09 | 0.002 | 0.103 |
|  | PROCESSING_OF_DNA_DOUBLE_STRAND_BREAK_ENDS | 1.97 | 0.003 | 0.103 | CITRIC_ACID_CYCLE_TCA_CYCLE | -2.07 | 0.003 | 0.103 |
|  | HOMOLOGOUS_DNA_PAIRING_AND_STRAND_EXCHANGE | 1.95 | 0.003 | 0.103 | NEUTROPHIL_DEGRANULATION | -2.04 | 0.001 | 0.103 |
|  | PHOSPHORYLATION_OF_EMI1 | 1.91 | 0.007 | 0.126 | GENERATION_OF_SECOND_MESSENGER_MOLECULES | -2.02 | 0.002 | 0.103 |
|  | RESOLUTION_OF_SISTER_CHROMATID_COHESION | 1.88 | 0.004 | 0.103 | PD_1_SIGNALING | -2.00 | 0.003 | 0.103 |
|  | G0_AND_EARLY_G1 | 1.87 | 0.011 | 0.154 | ANTIGEN_PRESENTATION_FOLDING_ASSEMBLY_AND_PEPTIDE_LOADING_OF_CLASS_I_MHC | -1.98 | 0.003 | 0.103 |
|  | UNWINDING_OF_DNA | 1.87 | 0.010 | 0.149 | METABOLISM_OF_ANGIOTENSINOGEN_TO_ANGIOTENSINS | -1.97 | 0.003 | 0.103 |
|  | HDR_THROUGH_SINGLE_STRAND_ANNEALING_SSA | 1.86 | 0.006 | 0.119 | GLUCONEOGENESIS | -1.94 | 0.003 | 0.103 |
|  | INITIATION_OF_NUCLEAR_ENVELOPE_NE_REFORMATION | 1.85 | 0.008 | 0.134 | PYRUVATE_METABOLISM_AND_CITRIC_ACID_TCA_CYCLE | -1.94 | 0.003 | 0.103 |
|  | COLLAGEN_CHAIN_TRIMERIZATION | 1.84 | 0.006 | 0.119 | CREATION_OF_C4_AND_C2_ACTIVATORS | -1.94 | 0.003 | 0.103 |
|  | IKBA_VARIANT_LEADS_TO_EDA_ID | 1.82 | 0.012 | 0.164 | BIOLOGICAL_OXIDATIONS | -1.93 | 0.001 | 0.103 |
|  | TRANSCRIPTIONAL_REGULATION_BY_E2F6 | 1.82 | 0.006 | 0.119 | INTERLEUKIN_10_SIGNALING | -1.92 | 0.003 | 0.103 |
|  | SIGNALING_BY_FGFR4_IN_DISEASE | 1.54 | 0.060 | 0.282 | SIGNALING_BY_PDGFR_IN_DISEASE | -0.81 | 0.731 | 0.910 |
|  |  |  |  |  |  |  |  |  |
| Wikipathway | CYTOPLASMIC_RIBOSOMAL_PROTEINS | 2.01 | 0.004 | 0.053 | CODEINE_AND_MORPHINE_METABOLISM | -2.41 | 0.002 | 0.035 |
| (Curated pathway members) | PURINE_METABOLISM | 1.98 | 0.002 | 0.042 | ELECTRON_TRANSPORT_CHAIN_OXPHOS_SYSTEM_IN_MITOCHONDRIA | -2.27 | 0.001 | 0.035 |
|  | DNA_IRDOUBLE_STRAND_BREAKS_DSBS_AND_CELLULAR_RESPONSE_VIA_ATM | 1.97 | 0.003 | 0.052 | GLUCURONIDATION | -2.22 | 0.002 | 0.035 |
|  | SUPRESSION_OF_HMGB1_MEDIATED_INFLAMMATION_BY_THBD | 1.94 | 0.002 | 0.042 | CONSTITUTIVE_ANDROSTANE_RECEPTOR_PATHWAY | -2.19 | 0.002 | 0.035 |
|  | SIGNAL_TRANSDUCTION_OF_S1P_RECEPTOR | 1.92 | 0.008 | 0.073 | PATHWAYS_IN_CLEAR_CELL_RENAL_CELL_CARCINOMA | -2.17 | 0.001 | 0.035 |
|  | ATM_SIGNALING_PATHWAY | 1.89 | 0.003 | 0.050 | GLYCOLYSIS_AND_GLUCONEOGENESIS | -2.15 | 0.001 | 0.035 |
|  | RETINOBLASTOMA_GENE_IN_CANCER | 1.81 | 0.004 | 0.053 | ESTROGEN_METABOLISM | -2.13 | 0.002 | 0.035 |
|  | CANONICAL_NFKB_PATHWAY | 1.71 | 0.018 | 0.129 | OXIDATIVE_PHOSPHORYLATION | -2.12 | 0.001 | 0.035 |
|  | THE_EFFECT_OF_PROGERIN_ON_THE_INVOLVED_GENES_IN_HUTCHINSONGILFORD_PROGERIA_SYNDROME | 1.64 | 0.019 | 0.129 | HUMAN_COMPLEMENT_SYSTEM | -2.09 | 0.001 | 0.035 |
|  | DNA_REPAIR_PATHWAYS_FULL_NETWORK | 1.64 | 0.004 | 0.053 | COMPLEMENT_ACTIVATION | -2.09 | 0.002 | 0.035 |
|  | GENE_REGULATORY_NETWORK_MODELLING_SOMITOGENESIS | 1.60 | 0.041 | 0.207 | ALLOGRAFT_REJECTION | -2.08 | 0.001 | 0.035 |
|  | COMMON_PATHWAYS_UNDERLYING_DRUG_ADDICTION | 1.58 | 0.032 | 0.176 | AMINO_ACID_METABOLISM | -2.03 | 0.001 | 0.035 |
|  | DNA_IRDAMAGE_AND_CELLULAR_RESPONSE_VIA_ATR | 1.58 | 0.004 | 0.053 | TCA_CYCLE_AKA_KREBS_OR_CITRIC_ACID_CYCLE | -2.01 | 0.002 | 0.035 |
|  | 22Q112_COPY_NUMBER_VARIATION_SYNDROME | 1.57 | 0.004 | 0.053 | PREGNANE_X_RECEPTOR_PATHWAY | -2.01 | 0.002 | 0.035 |
|  | DNA_REPLICATION | 1.57 | 0.019 | 0.129 | TCA_CYCLE_AND_DEFICIENCY_OF_PYRUVATE_DEHYDROGENASE_COMPLEX_PDHC | -2.00 | 0.002 | 0.035 |
|  | CHOLESTEROL_BIOSYNTHESIS_PATHWAY | 1.56 | 0.044 | 0.216 | TYROBP_CAUSAL_NETWORK | -1.99 | 0.001 | 0.035 |
|  | PROTEOGLYCAN_BIOSYNTHESIS | 1.54 | 0.056 | 0.249 | PROXIMAL_TUBULE_TRANSPORT | -1.99 | 0.001 | 0.035 |
|  | MIRNA_TARGETS_IN_ECM_AND_MEMBRANE_RECEPTORS | 1.52 | 0.048 | 0.227 | FARNESOID_X_RECEPTOR_PATHWAY | -1.98 | 0.002 | 0.035 |
|  | NUCLEOTIDE_METABOLISM | 1.51 | 0.061 | 0.256 | NANOMATERIALINDUCED_INFLAMMASOME_ACTIVATION | -1.98 | 0.002 | 0.035 |
|  | ROLE_OF_ALTERED_GLYCOLYSATION_OF_MUC1_IN_TUMOUR_MICROENVIRONMENT | 1.50 | 0.060 | 0.255 | ARYLAMINE_METABOLISM | -1.92 | 0.002 | 0.035 |
|  |  |  |  |  | VEGFAVEGFR2_SIGNALING_PATHWAY | -1.25 | 0.041 | 0.227 |
|  |  |  |  |  |  |  |  |  |
| c6 | RB_P107_DN.V1_UP | 1.89 | 0.005 | 0.110 | RELA_DN.V1_DN | -1.87 | 0.001 | 0.061 |
| (Transcriptional targets of oncogenic pathways) | HOXA9_DN.V1_DN | 1.43 | 0.036 | 0.230 | RPS14_DN.V1_UP | -1.84 | 0.001 | 0.061 |
|  | CAHOY_OLIGODENDROCUTIC | 1.37 | 0.035 | 0.230 | KRAS.LUNG.BREAST_UP.V1_DN | -1.70 | 0.003 | 0.083 |
|  | E2F3_UP.V1_UP | 1.34 | 0.035 | 0.230 | KRAS.600.LUNG.BREAST_UP.V1_DN | -1.64 | 0.001 | 0.061 |
|  |  |  |  |  | BCAT_GDS748_UP | -1.59 | 0.012 | 0.149 |
|  |  |  |  |  | LEF1_UP.V1_DN | -1.58 | 0.001 | 0.061 |
|  |  |  |  |  | TBK1.DN.48HRS_UP | -1.57 | 0.012 | 0.149 |
|  |  |  |  |  | CYCLIN_D1_KE_.V1_DN | -1.52 | 0.006 | 0.128 |
|  |  |  |  |  | MTOR_UP.N4.V1_UP | -1.52 | 0.002 | 0.083 |
|  |  |  |  |  | KRAS.BREAST_UP.V1_UP | -1.52 | 0.013 | 0.149 |
|  |  |  |  |  | KRAS.KIDNEY_UP.V1_DN | -1.51 | 0.012 | 0.149 |
|  |  |  |  |  | ALK_DN.V1_UP | -1.50 | 0.013 | 0.149 |
|  |  |  |  |  | KRAS.BREAST_UP.V1_DN | -1.47 | 0.018 | 0.181 |
|  |  |  |  |  | NFE2L2.V2 | -1.46 | 0.004 | 0.110 |
|  |  |  |  |  | KRAS.LUNG_UP.V1_UP | -1.46 | 0.019 | 0.182 |
|  |  |  |  |  | KRAS.50_UP.V1_UP | -1.45 | 0.050 | 0.242 |
|  |  |  |  |  | CYCLIN_D1_UP.V1_UP | -1.45 | 0.013 | 0.149 |
|  |  |  |  |  | KRAS.300_UP.V1_DN | -1.45 | 0.026 | 0.200 |
|  |  |  |  |  | NOTCH_DN.V1_DN | -1.45 | 0.012 | 0.149 |
|  |  |  |  |  | MTOR_UP.V1_UP | -1.44 | 0.015 | 0.156 |

This table includes gene sets with FDR<0.25 in addition to potential lenvatinib target pathways. If more than 20 pathways met this criterion, only top 20 pathways were shown according to NES.

HCC, hepatocellular carcinoma; NES, normalized enrichment score; FDR, false discovery rate.
